# Design of New BLE GAP Roles for Vehicular Communications

**DOI:** 10.3390/s24154835

**Published:** 2024-07-25

**Authors:** Antonio Perez-Yuste, Jordi Pitarch-Blasco, Felix Alejandro Falcon-Darias, Neftali Nuñez

**Affiliations:** 1 ETSI Sistemas de Telecomunicación, Universidad Politécnica de Madrid, 28031 Madrid, Spain; antonio.perez@upm.es (A.P.-Y.); jordi.pitarch@upm.es (J.P.-B.); f.fdarias@alumnos.upm.es (F.A.F.-D.); 2 Instituto de Energía Solar, Universidad Politécnica de Madrid, 28040 Madrid, Spain

**Keywords:** BLE, GAP roles, vehicle-to-everything, V2X, C-ITS, vehicle-to-infrastructure, V2I, vehicle-to-vehicle, V2V, vehicular networks

## Abstract

Bluetooth Low Energy (BLE) is a prominent short-range wireless communication protocol widely extended for communications and sensor systems in consumer electronics and industrial applications, ranging from manufacturing to retail and healthcare. The BLE protocol provides four generic access profile (GAP) roles when it is used in its low-energy version, i.e., ver. 4 and beyond. GAP roles control connections and allow BLE devices to interoperate each other. They are defined by the Bluetooth special interest group (SIG) and are primarily oriented to connect peripherals wirelessly to smartphones, laptops, and desktops. Consequently, the existing GAP roles have characteristics that do not fit well with vehicular communications in cooperative intelligent transport systems (C-ITS), where low-latency communications in high-density environments with stringent security demands are required. This work addresses this gap by developing two new GAP roles, defined at the application layer to meet the specific requirements of vehicular communications, and by providing a service application programming interface (API) for developers of vehicle-to-everything (V2X) applications. We have named this new approach ITS-BLE. These GAP roles are intended to facilitate BLE-based solutions for real-world scenarios on roads, such as detecting road traffic signs or exchanging information at toll booths. We have developed a prototype able to work indistinctly as a unidirectional or bidirectional communication device, depending on the use case. To solve security risks in the exchange of personal data, BLE data packets, here called packet data units (PDU), are encrypted or signed to guarantee either privacy when sharing sensitive data or authenticity when avoiding spoofing, respectively. Measurements taken and their later evaluation demonstrated the feasibility of a V2X BLE network consisting of picocells with a radius of about 200 m.

## 1. Introduction

Vehicle-to-infrastructure (V2I), vehicle-to-vehicle (V2V), and vehicle-to-everything (V2X) communications have become essential research topics due to the rise of autonomous vehicles and increased awareness of road safety risks. Countries are adopting various technologies for vehicular communications, including 4G and 5G cellular networks and Wi-Fi [1,2]. However, these technologies come with significant costs in terms of hardware requirements and energy efficiency. For instance, low-end 5G chipsets can be expensive and are not energy efficient. Wi-Fi, on the other hand, is a cheaper option and has some functionalities specifically developed for V2X based on the IEEE 802.11p standard [3]. However, it has some limitations compared to C-V2X and a power consumption of at least 60 mA, compared to nominal values of 15 mA in Bluetooth Low Energy (BLE) [1,4].

Bluetooth was introduced in 2000. It first appeared in mobile phones and desktop computers and was later moved to printers and laptops. Subsequent improvements, mainly recent low-energy versions, have led to an increase in the number of use cases and possibilities where this wireless communication protocol can be applied [4]. Among them, the application of Bluetooth to vehicular communications is an option to be seriously considered.

Comparing Bluetooth Low Energy (BLE) with other technologies employed for vehicular communications, BLE offers low-cost, mass-market, off-the-shelf devices, has low energy consumption, and is specifically designed for high-interference environments [5]. In addition, the versatility of the BLE stack protocol and its ability to be easily programmed mean that this wireless technology can be naturally integrated into many different types of devices, making the number of use cases increase year after year [6]. Moreover, BLE has a maximum gross data throughput of 2 Mbps, which is not a limiting factor for vehicular communications [7,8,9]. Finally, despite general concerns about Bluetooth with respect to range and consumption, both factors have been clearly overcome after version 5. BLE 5.0 and later releases allow for longer working time with small batteries and several types of energy harvesting techniques [10,11]. Moreover, its new LE-coded PHY provides much higher ranges (from 100 m to 1 km) compared to Bluetooth 4 and former versions, which enables its use in vehicular scenarios as described in this article [4].

Given the expected proliferation of wireless devices in the future European communications network of intelligent transport systems (C-ITS), the adoption of an energy-sustainable and cost-efficient solution from the outset is crucial [12,13].

A fully operational and cooperative ITS environment based on BLE wireless sensing technology, embedded in ITS components, is expected to improve the current C-ITS initiative in a way that has already been referred to by several authors [9,14,15,16,17]. The introduction of BLE wireless technology in vehicular communications may expand the C-ITS European ecosystem in two different directions: communication and sensing. To meet that goal, a BLE security framework to secure messages and the adaptation of BLE to ITS stack are necessary [18,19].

We advocate for the introduction of BLE as a complementary technology alongside mobile cellular (C-V2X) and Wi-Fi (ITS-G5) solutions for the European C-ITS platform. Our proposal, named ITS-BLE, is focused on the use of BLE as a short-range wireless technology for the safety-critical exchange of information between vehicles and from vehicles to road infrastructure (V2X).

Previous works have mainly focused on advertising information about traffic density or weather conditions with BLE [20,21]. However, they were undertaken without interference and using static devices. On the other hand, in [22], some dynamic tests with BLE devices were performed to obtain figures on data throughput and packets loss rate, but no final solution for V2X communications was provided.

Our contribution consists of designing two new BLE GAP roles, including the security dimension, and a service application programming interface (API) to allow for the development of V2X applications. A BLE GAP role defines a connection profile and manages how BLE devices interoperate each other. To demonstrate the feasibility of the solution, we have also developed an operative prototype based on the nRF5340dk board. Our work can be classified as part of layer 6 according to the 6-layer model (6LM) for the structured description and categorization of urban traffic and environment. This layer is characterized by the exchange of digital information between vehicles, infrastructure, and other devices related to vehicular communication [23,24,25].

This paper is organized as follows: Section 2 explains our proposal and compares it with other alternatives. In Section 3, we describe how we implemented the BLE devices to provide all the functionalities required for vehicular networks. Section 4 addresses the security problems related to these communications and how they can be solved. In Section 5, real application scenarios are explained, and the results in static and dynamic conditions are presented. Finally, Section 6 presents our final conclusions.

## 2. ITS-BLE Proposal

Within the cooperative intelligent transport systems (C-ITS) initiative, various technologies are being standardized for vehicular communications. They can be classified into two general groups: Wi-Fi and 5G.

Wi-Fi technology is based on the IEEE 802.11p protocol, and in Europe, it is known as ITS-G5. In the United States, the same technology is known as DSRC (dedicated short-range communications) or WAVE (wireless access in vehicular environment). DSRC is specifically tailored for vehicle-to-vehicle (V2V) communications within vehicular ad hoc networks (VANETs). These networks are crucial for supporting applications that improve road safety and traffic efficiency, and they do so without the need for high data rate transfers [3,26,27].

The introduction of 5G provides enhanced reliability, reduced latency, and greater bandwidth compared to earlier generations (such as LTE). For the efficient operation of the cellular vehicle-to-everything (C-V2X) in VANETs, 5G networks offer the necessary connectivity [28].

While not originally designed for vehicular communications, BLE stands out due to its simple and quick connection establishment mechanisms. BLE can complement or replace other V2V technologies (like DSRC) in inter-vehicle communication scenarios. Unlike Wi-Fi and 5G, BLE does not require the network to set up a connection before data transmission. BLE5 beacons eliminate the need for individual connections with each vehicle, resulting in lower latencies and a comparable communication range of about 200 m, as will be shown later.

**Figure 1:**
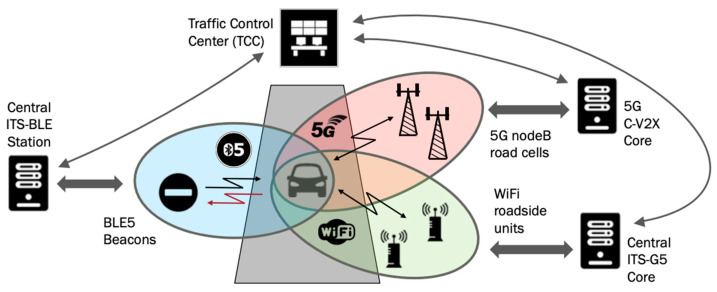
Schematic view about the coexistence of 5G, Wi-Fi, and BLE technologies for C-ITS.

In Figure 1, we can see an example of a C-ITS ecosystem with C-V2X, ITS-G5, and ITS-BLE technologies, coexisting all together, with some different uses depending on the situation.

To develop our BLE-based solution, first, we reviewed the functionality of the existing generic access profile (GAP) layer in the BLE protocol stack, and based on that, we designed two new GAP roles specifically adapted to vehicular scenarios, set up to control low-latency unidirectional and bidirectional connections in high-density environments. As a result, we have extended the present BLE functionality to enable vehicular-based applications, considering both security requirements and mobile vehicular communication needs.

## 3. BLE Contributions for ITS-BLE

**Figure 2:**
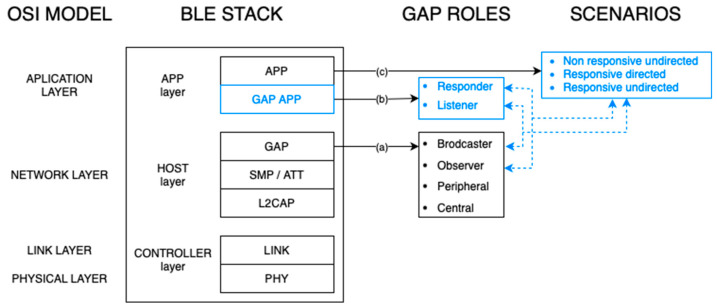
BLE protocol stack with our contributions in blue (ITS-BLE).

In the Bluetooth stack, we find three main layers: the controller, the host, and the application. In Figure 2, they have been represented alongside the typical OSI (open systems interconnection) architecture.

Host and controller layers are defined by the BLE SIG (special interest group) and cannot be modified. Therefore, all our contributions have been addressed to the application layer at top of the BLE Stack in Figure 2, which is divided into two smaller parts: (1) a GAP App sublayer, where we have defined the two new GAP App roles; and (2) an APP sublayer, where we have implemented some particular use cases. In Figure 2, the full new BLE stack is shown, with the already-exiting parts in black and our own contributions in blue.

In this section, we will start reviewing the current GAP roles that are defined in the BLE stack. According to Figure 2, this is marked as (a). With this in mind, we will compare the characteristics to see if we can use any of the existing ones for ITS-BLE. Then, we will see the other characteristics related to the addressability capabilities to see what we need for the ITS scenarios. After that, we will introduce our new GAP App roles, marked in Figure 2 as (b), as well as the resulting applications developed in the App sublayer, marked in Figure 2 as (c). These applications make use of the previous roles in (a) and (b) and integrate their own security elements, as will be explained later. Finally, we will collect statistics on packets correctly received with different configurations to verify the operation of our prototype.

### 3.1. Current GAP Roles

The generic access profile (GAP) layer is a crucial component of the BLE protocol stack. Its primary responsibility is to manage connections between devices. GAP ensures that all Bluetooth devices can establish baseband connections, regardless of the specific higher-level functionalities they support. Within the GAP layer, we find essential features related to modes, roles, advertising parameters, connection initiation, and security [7,29]. The four GAP roles defined in the Bluetooth core specification [30] are referred to in the next subsections.

#### 3.1.1. Broadcaster

The broadcaster role is optimized for transmit-only applications. Devices in this role periodically send out advertising packets (packet data units or PDUs) containing relevant data. Although typically associated with transmitters, the broadcaster role can also be assigned to devices that can receive data. For instance, a public hygrometer transmitting humidity values to all interested devices exemplifies the broadcaster role. This role leverages the link layer advertiser functionality.

#### 3.1.2. Observer

The observer role is used primarily by receiver-only applications. Devices in this role collect data from broadcast devices. Observers can also combine receiving capabilities with transmitting features. For instance, devices with displays can use the observer role to receive data from PDUs sent by broadcasting peers. The observer role relies on the link layer scanner functionality, and depending on the configuration, it can be either of the following:

- An active observer, which scans for advertising packets and may respond by sending scan requests to the broadcaster. This means that the active observer not only listens to advertisements but also actively seeks more information from the broadcasting device [31].
- A passive observer, which only listens for advertising packets without responding. It does not send any requests to the broadcaster and simply collects the advertising data passively [31].

#### 3.1.3. Peripheral

Peripheral roles operate at a subordinate level within the link layer. These devices use advertising packets to discover and connect with master peers. BLE’s resource-efficient design makes it ideal for edge deployments, minimizing processing memory and power requirements. This efficiency opens opportunities for low-cost BLE peripherals. The peripherals initiate connections with central devices (masters).

#### 3.1.4. Central

The central role corresponds to the link layer master. Central devices can establish multiple connections with slave peers. Asymmetric in nature, the central role requires greater computational resources compared to link layer slaves. Tablets and smartphones often play a central role in BLE networks. They listen for advertising packets from other devices and establish connections with selected devices. This process can be repeated to create mesh networks, connecting multiple devices within the same network.

In Table 1, we summarize the four GAP roles, highlighting their scannable and/or connectable capabilities. These roles determine whether devices engage in a three-way handshake connection or maintain a permanent round-trip connection.

### 3.2. PDU Addressability Capabilities

Additionally, BLE allows for the configuring of PDUs in two distinct ways [32]:

- Directed: These packets contain only the source and target Bluetooth addresses (MAC addresses) in the payload. They are like personalized messages sent directly to a recipient.
- Undirected: Any Bluetooth receiver can scan them. In undirected PDUs, packets can carry user data in the payload. It is like a public announcement that anyone can hear.

With these four GAP roles and two addressability capabilities, a wide range of applications can be built on top of the BLE stack. Some examples were shown in Table 1. However, they do not fit well for C-ITS scenarios; where low latency is demanded, high dense connectivity is required, and lightweight PDUs are needed.

These requirements can be met with the existing non-connectable roles in BLE, like broadcaster or passive observer. The problem is that they only support unidirectional communications. To overcome this constraint, we have developed two new GAP roles to support bidirectional communications with non-connectable BLE modes. The way to do that is by joining together two opposite unidirectional GAP roles into just a single one, as is explained next.

### 3.3. New GAP Application Roles

In terms of roles, each BLE device can be operated in one or more roles simultaneously. These roles impose specific restrictions and enforce behavioral requirements. Certain combinations of roles allow devices to communicate effectively. While roles are often associated with device types, their use cases can vary across different implementations [33].

To make BLE suitable for vehicular communications, we have designed two new extended GAP roles. They are operated in cooperation with the existing ones to make them more versatile. These new roles address specific requirements, related to the connection, allowing for bidirectional communication operating in a non-scannable and non-connectable configuration, and prioritizing security approaches for vehicular communications. The new GAP App roles here introduced are as follows.

#### 3.3.1. Scanner–Responder

The scanner–responder role is intended to be used by devices that combine the broadcaster GAP role with a scanning–advertising link layer role. The objective is to create a hybrid feature where a device captures and retains a PDU transmitted from a source. It then responds with its own PDU to that source or repeats the incoming PDUs to other targets.

By leveraging the existing broadcaster and observer roles, we also enhance security with PDU signing to ensure that data transmitted by BLE are not modified, as well as PDU decryption for receiving sensitive data.

#### 3.3.2. Advertiser–Listener

The advertiser–listener role is designed for devices that use the observer GAP role with an advertising–scanning link layer role. The objective is to enable a hybrid advertiser–listener feature, allowing a device to transmit its own PDU to other devices while capturing PDU responses from those peers.

Like the scanner–responder, in order to enhance security, we make use of signature checking to ensure that data received by any BLE are not modified, as well as PDU encryption for sending sensitive data.

### 3.4. Scenarios

**Figure 3:**
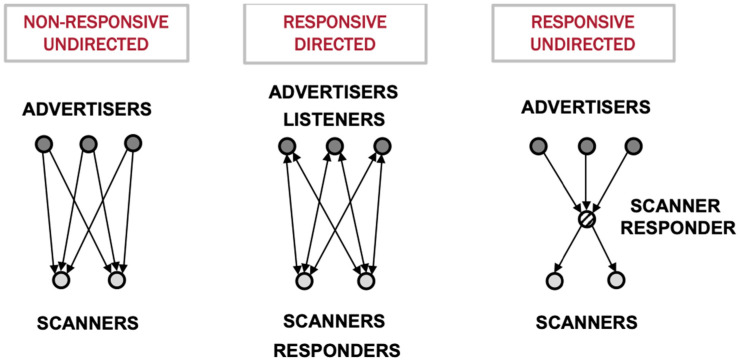
Scenarios considered in this paper for BLE vehicular communications.

We maintain the existing BLE addressability capabilities, unchanged. Our focus, instead, relies on leveraging two new GAP App roles: scanner–responder and advertiser–listener. These roles, combined with the established broadcaster and observer roles, along with our security layer, allow us to lay out various vehicular communication scenarios. Each scenario corresponds to a specific use case, as depicted in Figure 3.

#### 3.4.1. Scenario 1. Non-Responsive Undirected

In this scenario, we have a device with a broadcaster GAP role (undirected addressability) and another device with an observer GAP role. The resulting network resembles unidirectional broadcasting, where all scanners receive PDUs from every advertiser. A practical application is in roadside traffic signs, where they are made to act as broadcasters to provide their own information. Vehicles, acting as scanners, stay informed about traffic conditions or restrictions. Our security enhancements (PDU signing) make this setup ideal for implementing the European Intelligent Speed Assistance (ISA) system [34].

#### 3.4.2. Scenario 2. Responsive Directed

Here, we combine an advertiser–listener GAP App role (undirected addressability) with a scanner–responder GAP App role (directed addressability). The result is a bidirectional peer-to-peer network, with advertisers transmitting PDUs to all scanners. Scanners respond to each advertiser, which is listening to them. This configuration aligns well with open road tolling (ORT) systems [35]. Instead of traditional toll gates, advertisers inform passing vehicles about toll fees. Vehicles respond with their identification data, enabling seamless traffic management and fee charging without stopping or reducing velocity.

#### 3.4.3. Scenario 3. Responsive Undirected

In this scenario, we employ three types of BLE devices: devices with broadcaster GAP roles (undirected addressability), devices with scanner–responder GAP App roles (undirected addressability), and devices with observer GAP roles.

The resulting network forms a relay-broadcasting system, where scanners extend the range of advertisers. A practical use case could involve a traffic-light-controlled junction [36]. Close-approaching vehicles receive status updates, while vehicles further away rely on this information.

A relevant question which naturally arises is whether these three vehicular scenarios could be implemented by means of BLE devices with peripheral and central roles in a connectable configuration, and what advantages they offer compared to other alternatives such as Wi-Fi or 5G.

As we have seen before, something that is different in BLE compared to other technologies is that Bluetooth offers a native non-connectable configuration, which is perfect for vehicular scenarios where receivers must connect with different infrastructure beacons so often. In other technologies, to set up a connectable configuration requires some time. In 5G, for example, it would be around 1 to 5 ms [37], and in Wi-Fi, this time would be different depending on the environment [38]. These times include the detection of the peripheral and handshakes of keys. This action is conducted one-to-one; consequently, the delay is increased as more and more devices connect between each other in high-density scenarios, which are the most common in vehicular communications.

Let us transpose these times to a real vehicular scenario like, for example, a toll. Here, the toll would represent the master announcing its presence to every coming vehicle, which would be the slave. Consequently, every vehicle should compete to catch the master and start a connection as soon as possible to handshake data, while the rest of the vehicles must wait for their turn. In BLE, thanks to the non-connectable configuration, it is not necessary to wait for other vehicles to make their own transmission because, thanks to the flooding-like operation of the non-connectable mode in BLE, it is ensured that at least one packet reaches the destination in all cases.

### 3.5. Data Exchange

Let us next compare our non-connectable advertiser–listener and scanner–responder GAP App roles with the connectable central–peripheral GAP in standard BLE [29]. The aim is to demonstrate that for lightweight PDU communications, the first option is more efficient that the second one. We will use an ORT scenario as a use case.

**Figure 4:**
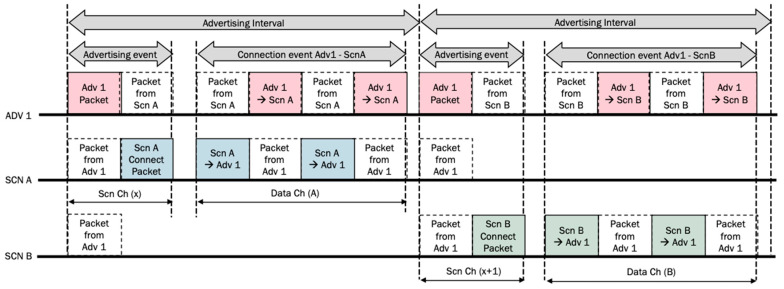
Example of an ORT use case with one toll and two vehicles in a connectable setup.

To guarantee a successful exchange of information when any impairment of the radio channel or any collision between packets comes up, an automatic repeat strategy will be followed. For the sake of comparison, let us consider that every PDU is sent twice. In the best case, that is, when every scanning window matches perfectly with the advertising interval, for one toll (Adv 1) and two vehicles (Scn A and Scn B), in a connectable setup, the timing of the packets would be as shown in Figure 4.

In that Figure, two advertising intervals were considered to receive twice the transmission of the PDU from the toll (Adv 1). In the first one, the first vehicle (Scn A) catches up the PDU, while the second vehicle (Scn B) catches up the PDU in the second advertising interval. Every time a connection event is started, the PDU of a vehicle is sent twice too. Due to BLE protocol, a paired PDU must be returned from the toll (Adv 1) to the vehicle.

**Figure 5:**
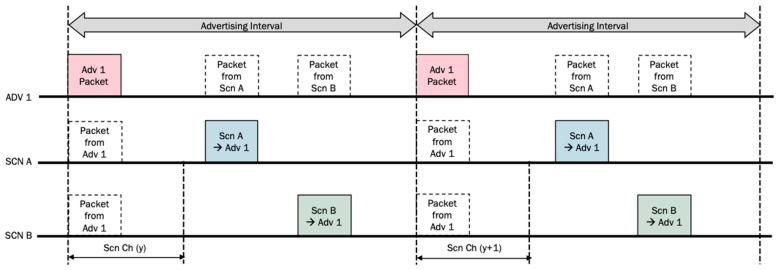
Example of an ORT use case with one toll and two vehicles in a non-connectable setup.

With a responsive directed setup, when the best case is considered again, that is, the scanning window always matches with the advertisement interval, the exchange of packets is made as simple as it is in Figure 5.

It can be seen how the toll packet (Adv 1) is received by both vehicles at the same time, so they can start the response process according to their respective advertising intervals. By its side, after transmitting, the toll is waiting for responses to be grabbed.

By analyzing Figure 4 and Figure 5, we can observe a lower channel occupancy and a higher efficiency for the non-connectable configuration when it is compared to the connectable setup. The red packets are sent by the advertiser and the blue and green ones are sent by different scanners.

Finally, we not only need to optimize the intervals for sending and receiving data; we also have to optimize the information transmitted inside our system. To that end, we have designed two types of messages, depending on the device. On the advertiser–listener side, we have designed a broadcasting PDU with a data payload length of 31 bytes. Information that we have included here are as follows: the company ID, the road ID, the site ID, orientation, time zone, easting, northing, TSSI, adv interval, and control information.

On the other side, in the scanner–responder device, we have designed a PDU with a data payload length of 31 bytes too, where the following information is included: the company ID, the plate, the time zone, the easting, the northing, TSSI, the interval, and the control information. Then, from only the data mentioned previously, we can ensure that all these packets have the smallest data required for any use case.

**Figure 6:**
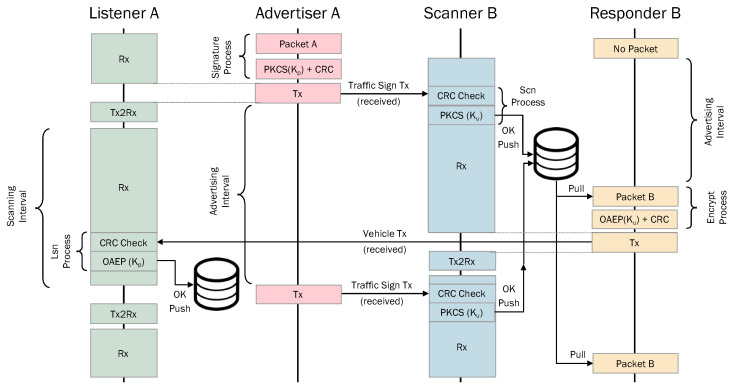
Flowchart of ITS-BLE proposal with the packets exchange between advertiser–listener and scanner–responder.

In Figure 6, we can see a flowchart of the packets transmitted between the advertiser–listener and the scanner–responder.

Both data payloads can be increased up to 255 bytes by using BLE extended adverting PDUs to include the PDU signature, in the case of an advertiser–listener device, and the encrypted PDU, in the case of a scanner-responder device. In next section, both security levels are duly explained.

## 4. Security Risks

When dealing with vehicular communications, we need to ensure that security risks are adequately addressed. There has been a relevant increase in security from classic Bluetooth [7] to BLE [39,40,41]. Even though, a BLE security framework was specifically developed at the App sublayer to ensure secure communications in vehicular scenarios, classifying security and privacy threads and researching real attacks that BLE can suffer to determine their implications.

We have devised two different approaches depending on the GAP App role. For an advertiser, a Bluetooth beacon sensor [17], which is profiled as a public BLE device, i.e., a traffic sign or a toll advertiser, privacy is not a sensitive issue. On the other hand, spoofing does represent a serious risk as they may reproduce fake traffic elements to distort the real information and create confusion in the best case or a threat in the worst case. The way to mitigate this problem is by introducing a signature in the PDU to guarantee its authenticity.

However, for a responder, which is profiled as a BLE private device, because the sensor is at the vehicle or pedestrian side, privacy turns into a truly important issue. In this case, PDUs can contain sensitive information about the vehicle or the user; thus, to mitigate risks, encryption has been chosen as the current method to address any potential security concerns.

**Figure 7:**
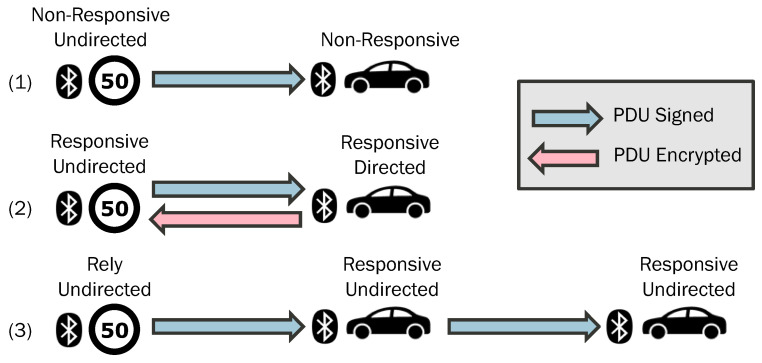
Security elements proposed for vehicular communication scenarios proposed in Section 3.

Figure 7 shows an application of these two security elements, i.e., authentication (draws as blue—PDU signed) and encryption (draws as red—PDU encrypted), for vehicular communication scenarios referred to in Section 3.

### 4.1. PDU Signing for a BLE Advertiser

For an advertiser, we have divided the PDU into two parts: one containing the clear message to be transmitted and the other containing the signature of the hash of the clear message. With this structure, one can not only check that the PDU has not been modified by a non-authorized third party, i.e., integrity; the authenticity of the PDU can be verified as well.

To obtain the hash of the clear message, a secure hash algorithm with a digest size of 256 bits, i.e., SHA-256, has been employed; while for signing the hash, an RSA-PKCS asymmetric encryption has been selected [42]. Consequently, the length of the payload of the PDU, in our application, was increased from 20 to 148 bytes, excluding header and error detection bytes. The private key was assigned to the advertiser, while the public key was given to the scanner.

At the scanner, the part of the received PDU with the clear message is processed to obtain the hash again, while the part of the PDU with the signed message is decrypted to recover the original hash. At this point, both hashes can be compared in between them; so, if no difference is found, this means that no hacking is taking place. On the other hand, if any difference is found between both hashes, this likely reveals a potential threat acting on the received PDU.

This security solution works due to the lack of sensitive information sent by the transmitter. Messages where the PDU is signed must be employed on non-confidential data and should be accessible to everyone around the transmitter. However, this would not be effective in other types of messages where the guarantee of privacy is the most important goal.

### 4.2. PDU Encryption for Sensitive Data

When sensitive data need to be shared, it is preferable to proceed with an encryption process instead of using a message-signing algorithm as in the previous example. In this way, potential threats such as eavesdropping can be avoided. Eavesdropping consists of listening to packages without the consent or authorization of the owner [36].

That is the usual case in a responder, where private information from a vehicle or a pedestrian is required to be securely shared with the listener. The approach is currently to use an asymmetric cryptography algorithm. The clear message in the responder is encrypted by means of a public key, while the listener recalls the message after decrypting with the corresponding private key. The algorithm employed for that purpose in our system is the RSA-OAEP scheme [43]. As a result, the length of the payload of the PDU in our application was increased from 20 bytes to 128 bytes.

These security solutions work properly if each user has their own public and private keys. While the first one can be known by everyone, the second one must only be known by the owner. With both keys, we can ensure that the system is secure for the requirements that are nowadays necessary in a vehicular communications network.

## 5. Evaluation of ITS-BLE for C-ITS Applications

This section explores the feasibility of implementing BLE for C-ITS applications. Leveraging the GAP roles and BLE addressability capabilities detailed in Section 3, we conducted a proof-of-concept study focusing on three representative use cases for V2I and V2V communication networks.

These use cases align well with the C-ITS services identified by the European Commission for the C-ITS platform [12,13]. As envisioned, the deployment of this platform will contribute to enhanced road safety, improved travel predictability and comfort, and a reduced environmental footprint [44].

### 5.1. C-ITS Scenarios

The selection of these use cases is based on our research criteria and does not limit the implementation of other services with respect to the proposed GAP App roles. Any vehicular communication service characterized by sporadic, bidirectional, and short data exchange could be approached with a similar methodology. To emphasize the integration of BLE sensors with security enhancements for this C-ITS application, we have adopted the name ITS-BLE.

#### 5.1.1. Intelligent Speed Assistance (ISA) with ITS-BLE

The European Union has proposed the use of on-board intelligent speed systems in vehicles to ensure that drivers are aware of and comply with speed limits [45]. These systems can ultimately adapt to dynamic speed limits based on traffic density or weather conditions. As automobile manufacturers increasingly integrate this technology, the potential use cases for ISA are likely to expand.

**Figure 8:**
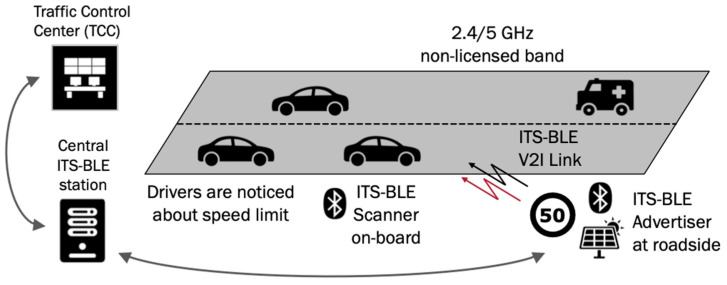
Schematic model of an ISA system.

Figure 8 depicts an electronic system leveraging ITS-BLE to prevent drivers from exceeding speed limits. This scenario utilizes the non-responsive undirected communication scheme detailed in Section 3. As shown by the arrows, messages are broadcasted within the network. Bidirectional communication is not required for this application. Therefore, the system can work with BLE devices operating in the broadcaster GAP role for traffic signs and in the observer GAP role for vehicles.

#### 5.1.2. Open Road Toll (ORT) with ITS-BLE

Traditional toll highways suffer from inefficiencies associated with vehicle identification and toll collection. Vehicles often encounter delays at toll booths or experience reduced speeds when passing through electronic toll collection systems. Only expensive and elaborate toll plazas with sophisticated image processing can achieve seamless, high-speed tolling [35].

**Figure 9:**
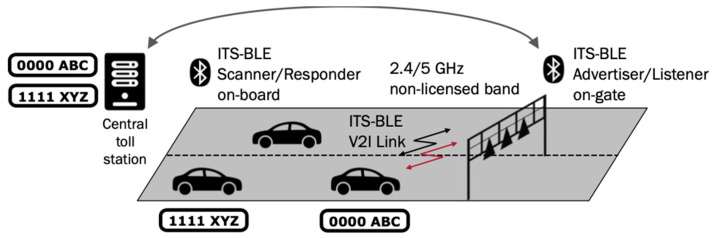
Schematic model of an ORT system.

This section proposes a cost-effective and easily deployable open road tolling (ORT) system, leveraging ITS-BLE technology. Figure 9 illustrates the implementation, which uses the responsive directed communication scheme detailed in Section 3. A bidirectional communication link is established between the ORT system infrastructure and vehicles traveling along the road, depicted by the two-way arrows. The data collected can be used for various purposes, including traffic statistics generation, infrastructure usage billing, and even driver compliance monitoring.

#### 5.1.3. Smart Traffic Light (STL) with ITS-BLE

The preceding use cases exemplified V2I communication with a range limitation determined by the BLE device itself. This section explores a scenario involving a traffic light at a road junction. Here, information is disseminated to nearby vehicles, which can then act as relays to extend the system’s reach. In essence, a vehicle first observes the traffic light data and then retransmits them to increase the overall coverage. This scenario uses the responsive undirected communication scheme detailed in Section 3.

**Figure 10:**
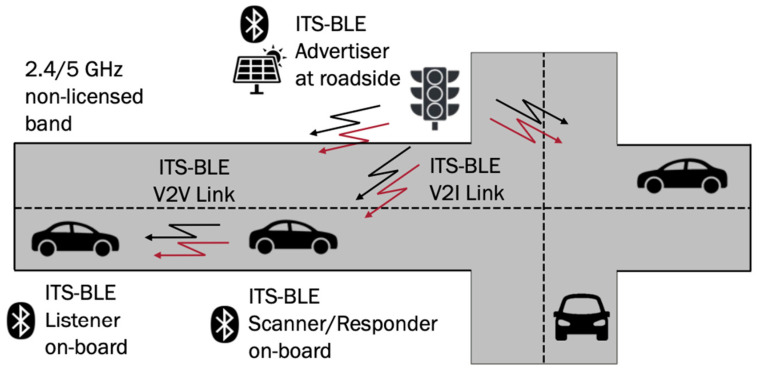
Schematic model of a STL system.

Figure 10 depicts a schematic representation of a BLE-based traffic light control system for a road junction. Vehicles closest to the traffic light receive and relay the PDU containing traffic light information to vehicles further away. To ensure efficiency, a limit must be imposed on the number of hops each PDU can undergo. Traffic light data become increasingly irrelevant for vehicles located progressively further from the junction.

The arrows in Figure 10 illustrate message transmissions between infrastructure and vehicles. This scenario assumes a single hop, and the PDU payload varies depending on the approaching direction (lane) to ensure that relevant information reaches the intended recipients.

### 5.2. Measurements Results

To evaluate the feasibility of our BLE-ITS system under various conditions and to compare its performance with other wireless technologies used for similar purposes, we conducted a series of tests. These tests included static scenarios to assess the maximum range under real conditions, as well as real-world tests with moving BLE sensors to evaluate packet reception rates.

The first test involves a static outdoor environment without movement. We configured the advertiser and listener intervals at 150 ms and 100 ms, respectively, aiming to determine the system’s range in this controlled setting. We chose these intervals due to the requirements in 3GPP, where our ITS scenarios would be inside the low-latency class, which needs values of around 100 ms [46]. The transmission time is 40 s; so, a total amount of 260 packets can be sent in the best case. Tests employed a transmission power of 3 dBm with GW.26.0112 antennas (Taoglas, Ireland) and Nordic nRF5340dk boards (Nordic Semiconductor, Trondheim, Norway), for both the advertiser–listener and the scanner–responder devices. Additional information on the BLE range and external characterization can be found in our previous works [47,48].

**Figure 11:**
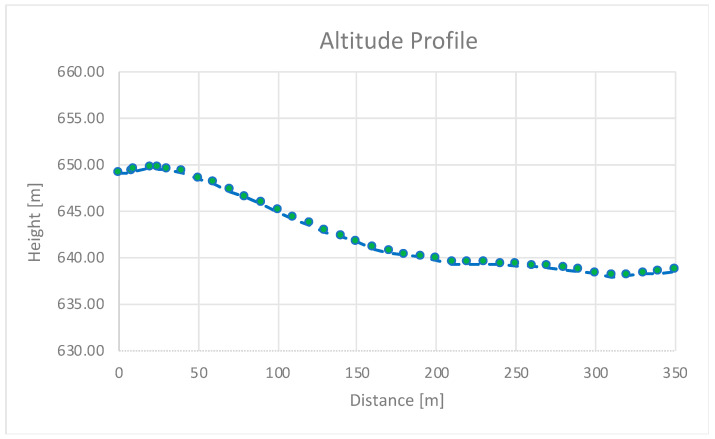
Altitude profile of the road where tests were conducted.

As for the test, it was carried out in an outdoor scenario along a road with changing altitude. Figure 11 shows the altitude profile of the road where the static test was executed. If we go through it carefully, it can be observed that the line of sight between transmitter and receiver is lost for distances of around 150 to 200 m. Even though, it was observed that the system also worked well in non-line-of-sight conditions, obtaining ranges over 300 m. We conducted the test with an outside temperature of 25 degrees.

**Figure 12:**
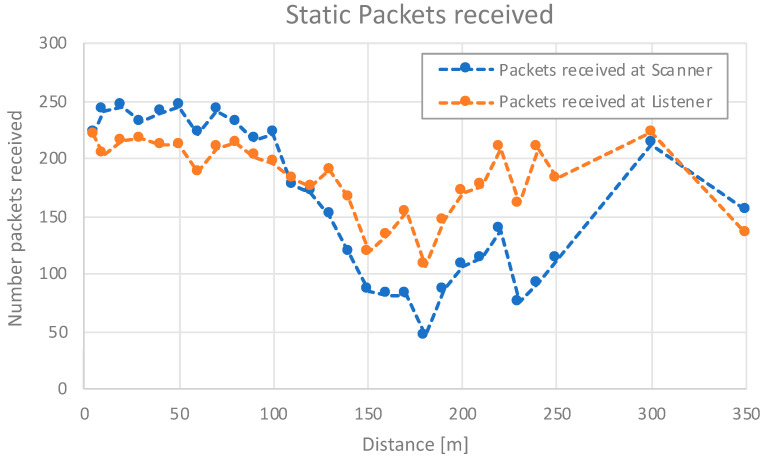
Static packets received in both directions at both devices in the static scenario.

Figure 12 depicts the results. PDU reception ceased at 350 m, exceeding the practical range requirements of our system. These results demonstrate that BLE offers a significant range advantage compared to other wireless technologies like Wi-Fi (150 m) or Bluetooth 4 (150 m) [49,50]. The high packet reception rate here obtained ensures reliable message delivery even in non-line-of-sight conditions.

The latency for these vehicular communications is 150 ms for the scanner and 100 ms for the listener, which has been selected after trying with smaller values which did not provide better results, and the consumption was higher because the need to send more packets. The final parameters selected for the experiment meet the low-latency class requirements, i.e., values of around 100 ms [46]. The power consumption obtained values under 15 mA [4,51].

**Figure 13:**
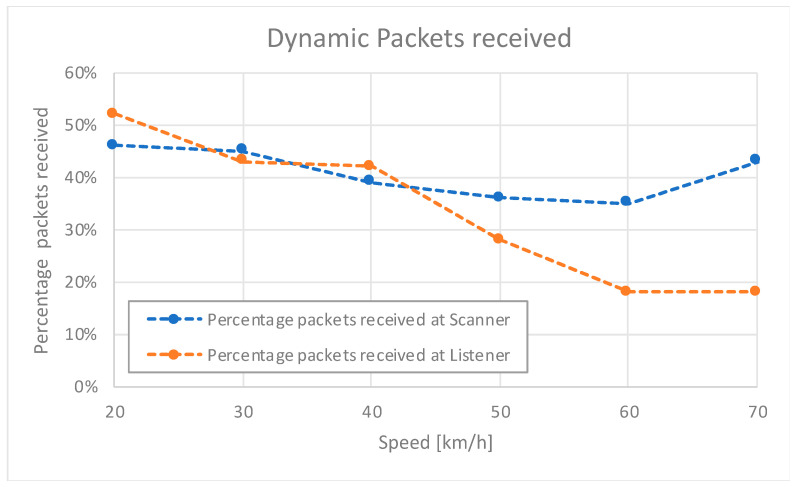
Percentage of dynamic packets received in both directions at both devices in the dynamic scenario.

Based upon the promising range results observed in the static scenario, we further evaluated the system performance in a dynamic scenario. This test involved a vehicle traveling at various speeds, and we measured the corresponding packet reception rates. Figure 13 depicts the packet reception results at different speeds.

Tests were conducted with the advertiser–listener at the roadside and the scanner–responder on board a vehicle traveling from far away towards the advertiser–listener. The account of received packets started as soon as the scanner–responder device received the first one, and it finished once the vehicle passed by the roadside advertiser–listener, or when the test time exceeded 40 s.

As expected, Figure 13 demonstrates a direct correlation between speed and the percentage of packets received at the listener due to the fact that the responder is always sending the same number of packets; that is, lower speeds lead to higher rates of received packets due to the longer time spent within the BLE system range. On the other hand, the scanner is always receiving at a certain rate, while packets sent by the advertiser are not limited, thus obtaining approximately the same percentage of packets no matter the speed.

Based on the findings in both static and dynamic scenarios, we conclude that our ITS-BLE system provides enough range and redundancy for vehicular communications, leveraging our adapted GAP App roles and effectively facilitating communications across the scenarios formerly defined for C-ITS applications.

## 6. Conclusions

The ITS-BLE system proposed in this paper takes advantage of newly designed GAP App roles with enhanced security features to demonstrate their promising capabilities with respect to V2I and V2V communication in C-ITS applications. The system offers several advantages over existing solutions. The main ones are as follows:

- Cost-Effectiveness: BLE technology utilizes low-cost hardware compared to dedicated V2X solutions such as 5G. This facilitates the broader adoption and deployment of various C-ITS services.
- Scalability: The non-connectable configuration of BLE devices eliminates the need for complex connection management, especially in high-density vehicular environments.
- Security: The proposed security framework, which incorporates PDU signing and encryption, safeguards against unauthorized access, message tampering, and eavesdropping.
- Range: BLE offers a significant range advantage compared to Wi-Fi or Bluetooth 4, i.e., from 150 m to more than 350 m, as demonstrated in the static test results. This extended range has proven to be sufficient for various C-ITS use cases.
- Efficiency: The non-connectable configuration eliminates connection delays experienced in traditional connectable setups. This is crucial for time-sensitive V2X communication scenarios such as ORT systems. The latency for BLE vehicular communications is 150 ms for the scanner and 100 ms for the listener, which were selected for our system after different trials. The power consumption obtained values under 15 mA.
- Flexibility: The newly developed adaptable GAP App roles (scanner–responder and advertiser–listener) meet the requirements of diverse V2I and V2V communication, including unidirectional and bidirectional data exchange.

Results of the dynamic test showcase the system reliability in maintaining communication even at varying vehicle speeds from 20 to 70 km/h. This robustness ensures effective information dissemination within the BLE range.

Overall, the ITS-BLE system presents a compelling solution for C-ITS applications. Its cost-effectiveness, scalability, security, range, efficiency, and flexibility make it a viable alternative to existing technologies. Future research can explore further optimization of the system’s performance and investigate its integration with other C-ITS infrastructure components for a more efficient connected transportation ecosystem.

## Figures and Tables

**Table 1:** Summary of current BLE GAP roles and their ability to be scannable or connectable. One example of application per role is provided.

GAP Roles	Advertiser	Scanner	Application
Broadcaster	Non-connectable and non-scannable	n/a	Beacon
Observer (passive)	n/a	Non-connectable and non-scannable	Monitoring
Observer (active)	Scannable	Scannable	Paging
Peripheral	Connectable	n/a	Slave (headset)
Central	n/a	Connectable	Master (smartphone)

n/a: not applicable.

## Data Availability

Data are contained within the article.

## Abbreviations

Acronyms in this paper and their full form are shown below.

API	Application Programming Interface	ORT	Open Road Tolling
BLE	Bluetooth Low Energy	OSI	Open Systems Interconnection
C-ITS	Cooperative ITS	PDU	Packed Data Unit
C-V2X	Cellular V2X	SIG	Special Interest Group
DSRC	Dedicated Short-Range Communications	STL	Smart Traffic Light
GAP	Generic Access Profile	V2I	Vehicle-to-Infrastructure
ISA	Intelligent Speed Assistance	V2V	Vehicle-to-Vehicle
ITS	Intelligent Transport System	V2X	Vehicle-to-All
ITS-G5	ITS in 5 GHz Band	VANET	Vehicular Ad Hoc Networks
LTE	Long-Term Evolution	WAVE	Wireless Access in Vehicular Environment

## References

[1] Mannoni V., Berg V., Sesia S., Perraud E. A Comparison of the V2X Communication Systems: ITS-G5 and C-V2X. Proceedings of the IEEE 89th Vehicular Technology Conference (VTC2019-Spring).

[2] Kotsi A., Mitsakis E., Tzanis D. Overview of C-ITS deployment projects in Europe and USA. Proceedings of the IEEE 23rd International Conference on Intelligent Transportation Systems (ITSC).

[3] (2010). IEEE Standard for Information Technology–Local and Metropolitan Area Networks–Specific Requirements—Part 11: Wireless LAN Medium Access Control (mac) and Physical Layer (PHY) Specifications Amendment 6: Wireless access in Vehicular Environments.

[4] Argenox Introduction to Bluetooth Low Energy (BLE). https://www.argenox.com/library/bluetooth-low-energy/introduction-to-bluetooth-low-energy-v4-0/.

[5] Bronzi W., Frank R., Castignani G., Engel T. (2016). Bluetooth Low Energy performance and robustness analysis for inter-vehicular communications. Ad Hoc Netw..

[6] Jeon K.E., She J., Soonsawad P., Ng P.C. (2018). BLE beacons for internet of things applications: Survey, challenges, and opportunities. IEEE Internet Things J..

[7] Zeadally S., Siddiqui F., Baig Z. (2019). 25 years of Bluetooth technology. Future Internet.

[8] Natgunanathan I., Fernando N., Loke S.W., Weerasuriya C. (2023). Bluetooth Low Energy Mesh: Applications, Considerations and Current State-of-the-Art. Sensors.

[9] García-Ortiz J.C., Silvestre-Blanes J., Sempere-Payá V. (2021). Experimental Application of Bluetooth Low Energy Connectionless in Smart Cities. Electronics.

[10] Sanislav T., Mois G.D., Zeadally S., Folea S.C. (2021). Energy harvesting techniques for internet of things (IoT). IEEE Access.

[11] Bouhedma S., Bin-Taufik J., Lange F., Ouali M., Seitz H., Hohlfeld D. (2024). Different Scenarios of Autonomous Operation of an Environmental Sensor Node Using a Piezoelectric-Vibration-Based Energy Harvester. Sensors.

[12] European Commission (2016). A European Strategy on Cooperative Intelligent Transport Systems, a Milestone towards Cooperative, Connected and Automated Mobility.

[13] C-ITS Platform—Final Report January 2016 Cites and Regions for Transport Innovation. https://www.polisnetwork.eu/wp-content/uploads/2019/09/c-its-platform-final-report-january-2016.pdf.

[14] Rocha D., Teixeira G., Vieira E., Almeida J., Ferreira J. (2023). A Modular In-Vehicle C-ITS Architecture for Sensor Data Collection, Vehicular Communications and Cloud Connectivity. Sensors.

[15] Friesen M.R., McLeod R.D. (2015). Bluetooth in intelligent transportation systems: A survey. Int. J. Intell. Transp. Syst. Res..

[16] Thomas K., Fouchal H., Cormier S., Rousseaux F. Intelligent transport system based on Bluetooth. Proceedings of the Communication Technologies for Vehicles: 14th International Workshop, Nets4Cars/Nets4Trains/Nets4Aircraft 2019.

[17] Guerrero-Ibáñez J., Zeadally S., Contreras-Castillo J. (2018). Sensor technologies for intelligent transportation systems. Sensors.

[18] Contreras-Castillo J., Zeadally S., Guerrero-Ibáñez J.A. (2017). A seven-layered model architecture for Internet of Vehicles. J. Inf. Telecommun..

[19] Molina E., Rios R., Agudo I. An empirical evaluation of BLE for ITS scenarios. Proceedings of the IEEE 98th Vehicular Technology Conference (VTC2023-Fall).

[20] Barba C.T., Mateos M.A., Soto P.R., Mezher A.M., Igartua M.A. Smart city for VANETs using warning messages, traffic statistics and intelligent traffic lights. Proceedings of the 2012 IEEE Intelligent Vehicles Symposium.

[21] García J.C., Silvestre-Blanes J., Sempere-Paya V., Ponce R. Feasibility of Bluetooth 5.x connectionless communications for I2V applications. Proceedings of the 25th IEEE International Conference on Emerging Technologies and Factory Automation.

[22] Aza A., Melendi D., García R., Pañeda X.G., Pozueco L. (2022). Bluetooth 5 performance analysis for inter-vehicular communications. Wireless Netw..

[23] Scholtes M., Westhofen L., Turner L.R., Lotto K., Schuldes M., Weber H., Wagener N., Neurohr C., Bollmann M., Körtke F. (2021). 6-Layer Model for a Structured Description and Categorization of Urban Traffic and Environment. IEEE Access.

[24] Weber H., Glasmacher C., Schuldes M., Wagener N., Eckstein L. Holistic Driving Scenario Concept for Urban Traffic. Proceedings of the 2023 IEEE Intelligent Vehicles Symposium (IV).

[25] Stepanyants V., Romanov A. An Object-Oriented Approach to a Structured Description of Machine Perception and Traffic Participant Interactions in Traffic Scenarios. Proceedings of the 2022 IEEE 7th International Conference on Intelligent Transportation Engineering (ICITE).

[26] Kelarestaghi K.B., Foruhandeh M., Heaslip K., Gerdes R. (2019). Survey on vehicular ad hoc networks and its access technologies security vulnerabilities and countermeasures. arXiv.

[27] Hendaoui M. Integration of Wi-Fi, VLC, WiMAX, 4G, and 5G Technologies in Vehicular Ad Hoc Networks: A Comprehensive Review. Proceedings of the 2023 7th International Symposium on Innovative Approaches in Smart Technologies (ISAS).

[28] Askar S., Qadir G.A., Rashid T.A. (2021). SDN Based 5G VANET: A review. Int. J. Sci. Bus..

[29] Bluetooth Special Interest Group (2023). Bluetooth Core Specification, Version 5.4. https://www.bluetooth.com.

[30] Townsend K., Cufí C., Davidson R. (2014). Getting Started with Bluetooth Low Energy.

[31] Understanding the Generic Access Profile (GAP) Qualcomm Developer Network. https://developer.qualcomm.com/hardware/qca4020-qca4024/learning-resources/understanding-generic-access-profile.

[32] Afaneh M. (2018). Advertising and scanning. Intro to Bluetooth Low Energy.

[33] Dian F.J., Yousefi A., Lim S. A practical study on Bluetooth Low Energy (BLE) throughput. Proceedings of the IEEE 9th Annual Information Technology, Electronics and Mobile Communication Conference (IEMCON).

[34] Ryan M. Intelligent Speed Assistance Technologies: A review. Proceedings of the ITRN2019.

[35] Bandi K., Shailendra S., Varanasi C. (2023). CV2X-PC5 Vehicle-Based Tolling Transaction System. Proc. IEEE Open J. Intell. Transp. Syst..

[36] Rai S.C., Nayak S.P., Acharya B., Gerogiannis V.C., Kanavos A., Panagiotakopoulos T. (2023). ITSS: An Intelligent Traffic Signaling System Based on an IoT Infrastructure. Electronics.

[37] Naragund J.G., Vijayalakshmi M., Kanakaraddi S.G. Fuzzy Controller for Traffic Management in 5G Networks. Proceedings of the IEEE 15th International Conference on Industrial and Information Systems (ICIIS).

[38] Abideen M.Z., Htut A.M., Aswakul C. Development of Control-Plane Switch Migration Testbed Using Mininet-WiFi for Software-Defined Vehicular Network. Proceedings of the 20th International Joint Conference on Computer Science and Software Engineering (JCSSE).

[39] Barua A., Al-Alamin M.A., Hossain M.S., Hossain E. (2022). Security and privacy threats for Bluetooth Low Energy in IoT and wearable devices: A comprehensive survey. IEEE Open J. Commun. Soc..

[40] Ghori M.R., Wan T.C., Sodhy G.C. (2020). Bluetooth Low Energy mesh networks: Survey of communication and security protocols. Sensors.

[41] Lacava A., Zottola V., Bonaldo A., Cuomo F., Basagni S. (2022). Securing Bluetooth Low Energy networking: An overview of security procedures and threats. Comput. Netw..

[42] Kocher P.C., Koblitz N. (1996). Timing Attacks on Implementations of Diffie-Hellman, RSA, DSS, and Other Systems. Advances in Cryptology—CRYPTO’96.

[43] Pointcheval D. (2002). How to Encrypt Properly with RSA. CryptoBytes.

[44] C-ITS Deployment Group Webpage. https://c-its-deployment-group.eu.

[45] Huysamen K., Collins M., Wardle A. (2021). General Safety Regulation, Technical Study to Assess and Develop Performance Requirements and Test Protocols for Various Measures Implementing the New General Safety Regulation, for Accident Avoidance and Vehicle Occupant, Pedestrian and Cyclist Protection in Case of Collision.

[46] Boban M., Kousaridas A., Manolakis K., Eichinger J., Xu W. (2018). Connected Roads of the Future: Use Cases, Requirements, and Design Considerations for Vehicle-to-Everything Communications. IEEE Veh. Technol. Mag..

[47] Perez-Yuste A., García J.S., Serrano C.R., Blanco-Archilla Y. Low-cost Radio Channel Sounder for the ISM 2.4 GHz Band. Proceedings of the 2022 IEEE Conference on Antenna Measurements and Applications (CAMA).

[48] Perez-Yuste A., Pachacama J.L., García J.S. Characterization of the 2.4 GHz-band using a semiempirical model and a ray tracing model. Proceedings of the 2023 IEEE Conference on Antenna Measurements and Applications (CAMA).

[49] Jackson B., Nisha A.S.A., Varalakshmi S., Darwin N., Varun M. Enhancing WiFi Range and Throughput with Beam Steering Antennas. Proceedings of the 2023 7th International Conference on Electronics, Communication and Aerospace Technology (ICECA).

[50] Decuir J. (2010). Bluetooth 4.0: Low Energy.

[51] Nordic Semiconductor (2022). nRF5340 DK Hardware—User Guide.

